# Factors related to a successful professional development for specialist nurses in surgical care: a cross-sectional study

**DOI:** 10.1186/s12912-023-01258-0

**Published:** 2023-03-22

**Authors:** Jenny Drott, My Engström, Eva Jangland, Victoria Fomichov, Marlene Malmström, Jenny Jakobsson

**Affiliations:** 1 Division of Nursing Science, Department of Health, Medicine and Caring Sciences, Linköping University, Linköping, Sweden; 2 Department of Surgery, Linköping University Hospital, Linköping, Sweden; 3 Institute of Health and Care Sciences, Sahlgrenska Academy, University of Gothenburg, Gothenburg, Sweden; 4 Department of Surgery Sahlgrenska, Region Västra Götaland, Sahlgrenska University Hospital, Gothenburg, Sweden; 5 Department of Surgical Sciences, Nursing Research, Uppsala University, Uppsala, Sweden; 6 Unit for Public Health and Statistics, County Council of Östergötland, Linköping University, Linköping, Sweden; 7 Department of Health Sciences, Lund University, Lund, Sweden; 8 Department of Surgery and Gastroenterology, Skåne University Hospital, Malmö, Sweden; 9 Department of Care Science, Faculty of Health and Society, Malmö University, Malmö, Sweden

**Keywords:** Clinical competence, Leadership, Professional competence, Surgical nursing, Working condition

## Abstract

**Background:**

A high level of competence among staff is necessary for providing patient-safe surgical care. Knowledge regarding what factors contribute to the professional development of specialist nurses in surgical care and why they choose to remain in the workplace despite high work requirements is needed. To investigate and describe the organizational and social work environment of specialist nurses in surgical care as part of studying factors that impact on professional development.

**Method:**

This was a cross-sectional study with a strategic convenience sampling procedure that recruited 73 specialist nurses in surgical care in Sweden between October to December 2021. The study was guided by STROBE Statement and checklist of cross-sectional studies. The validated Copenhagen Psychosocial Questionnaire was used, and additional demographic data. Descriptive statistics were performed and the comparison to the population benchmarks was presented as the mean with a 95% confidence interval. To study potential differences among the demographic and professional characteristics, pairwise t tests were used with Bonferroni adjustment for multiple comparisons with a significance level of 5%.

**Results:**

Five domains were identified as factors related to success, as they received higher scores in relation to population benchmarks: quality of leadership, variation of work, meaning of work and work engagement as well as job insecurity. There was also a significant association between a having a manager with low nursing education and job insecurity (*p* = 0.021).

**Conclusions:**

Quality of leadership is important for the professional development of specialist nurses in surgical care. Strategic work seems to include managers with a higher nursing education level to prevent insecure professional working conditions.

## Background

Today, many surgical clinics internationally are struggling with cost savings and nursing shortages, which is a major challenge for nursing management. In 2020, the World Health Organization (WHO) published a synthesis of contemporary evidence based on the important responsibilities of nurses and nurse management [1]. A high level of competence and well-educated nurses is necessary for providing patient-safe care [2]. It has been reported that there is a shortage of specialist nurses globally [1, 2]. Reversing this negative trend requires changes that create good conditions for high-quality surgical nursing and the opportunity for job satisfaction and professional development [3]. Therefore, more knowledge is needed about what factors contribute to the professional development of specialist nurses in surgical care and why they chose to remain in the workplace despite high work requirements.

Specialist nurses in surgical care have the competence to bridge the gap between nursing surgical research and implementation into clinical practice. In-depth competence leads to the needs of surgical patients being correctly addressed due to the provision of high-quality care and entails increased patient safety. Unfortunately, high employee turnover in the surgical context has had a negative impact on these aspects. At the same time, there has been considerable focus on why nurses and specialist nurses choose to leave employment. It appears to be generally accepted that the main reason is high workload combined with low salary. This is in line with previous findings, showing that a low salary and a lower degree of autonomy were strong predictors of specialist nurses leaving their jobs within a five-year period [4]. However, it has also been suggested that low job satisfaction is a reason for leaving employment [5, 6]. Perceived job satisfaction due to e.g., respect from colleagues and a possibility of continuous learning, is reasons to stay and used nursing professional skills to the fullest [7].

There are several underlying factors related to a nurse’s sense of job satisfaction and motivation, which can be divided into external and internal rewards. External rewards, such as salary, relationships with colleagues and opportunities for professional development, can be a driving force in nurses' work [4, 8]. Using one's skills and being independent in work as a nurse has also been shown to contribute to a sense of job satisfaction, which are examples of internal rewards [5, 9–11].

Previous studies have shown that there is a multifactorial impact on job satisfaction and professional development. Job satisfaction as a type of affective reaction to work that includes values, expectations and needs to thrive [12]. Furthermore, the motivation to work affects one's actions, what choices one makes and the degree of effort. It affects the acquisition of skills, professional development and how competence is utilized [13, 14].

One way to achieve professional development is by adapting goal-driven management, which means that the leadership and the organization actively work to achieve goals, such as quality indicators and feedback of quality in set goals. This may increase efficiency since professionals can influence their work situations and development and have their own responsibility to improve their working environment [14, 15]. Another successful model in the nursing context is the Magnet model, which originates from the United States. The Swedish Nurses' Association describes that in Magnet model management, nurses need to be included in mandates (Chief Nurse Officers) and participate in making decisions that affect the organization's visions, goals, and priorities [16]. To demonstrate a measurable outcome of developed nursing leadership, nurses must perceive that their efforts are valuable and that they are supported in their work. Magnet hospitals are characterized by, among other aspects, a high level of competence [17, 18].

Determining optimal staffing requirements is a complex issue. Currently, several workforce planning and modelling tools exist, which investigate matching patient needs and service requirements with required nursing numbers and competencies [19]. The theoretical Job Demands-Resources Theory (JD-R) describe the relationship between job demands and job resources, in line with mission-relevant staffing levels and the Magnet Model [18, 20]. Job demands are physical, social or organizational aspects of work that require constant physical or mental efforts on the part of the worker. Specialized nurses in surgical care are often involved in many technical tasks, including independent decision-making, administrative work and providing care for a large number of patients with serious medical conditions. Job resources, on the other hand, involve physical, psychological, social or organizational aspects of the work that can work in part to achieve work-related goals and to stimulate professional development. An imbalance between work requirements and work resources can, in accordance with the JD-R model, have organizational consequences, as this can lead to exhaustion, reduced motivation and the risk of nurses choosing to leave the workplace and, in the worst case, the profession [21, 22].

A shortage of nurses leads to a deterioration in the quality of care and loss of care, which in turn has an impact on patient safety [23–26]. It may be problematic to further develop surgical nursing over time without competence, and patients are often the ones who suffer. Reversing a negative trend requires changes that create opportunities for professional development and, consequently, good conditions for high-quality surgical nursing. A high level of competence among nurses is necessary for providing patient-safe surgical care. Therefore, more knowledge is needed about what factors contribute to the professional development of specialist nurses in surgical care and why they choose to remain in the workplace despite high work requirements.

The aim of this study was to investigate and describe the organizational and social work environment of specialist nurses in surgical care as part of studying the factors that impact on professional development. Research questions: What factors are related to success and less success in relation to the Swedish population benchmarks? Are there significant differences between factors and demographic variables?

## Methods

### Design

This was a cross-sectional study, guided by STROBE Statement and checklist of cross-sectional studies [27].

### Data collection

With a strategic convenience sampling procedure, specialist nurses in surgical care were recruited to the study between October to December 2021. The study was performed in Sweden and recruited specialist nurses in Swedish surgical care (in different public hospitals). The oral and written information about the study and reminders were disseminated via the National Association of Nurses in Surgical Care (NFSK) webpage and social media with a link to the questionnaire. The total members at this point in time (October 2021) were 191 specialist nurses and 5 nurse practitioners in surgical care. However, it is not known what percentage of NFSK members are active in social media, so the response rate was difficult to calculate. Since the study population was a convenience sample, a post hoc power analysis was performed, and the study was adequately powered (see Data analysis).

### Measurement

The validated Copenhagen Psychosocial Questionnaire 3 (COPSOQ) was used in the study [28]. The research team distributed the COPSOQ as an anonymous digital web-based questionnaire. No changes were made to the questionnaire's items when they were transferred to a web-based questionnaire. In the survey, questions about demographic variables were asked in addition to the COPSOQ items and included the variables of sex, age, marital status, and education level. Additionally, questions were asked about professional experience as a registered nurse and specialist training, the number of jobs in which they had worked as a nurse and the number of years at the current workplace. The COPSOQ is a generic, theoretically based instrument that measures different aspects of the organizational and social work environment. The instrument was developed in Denmark in the early 2000s. Since then, the instrument has been updated and translated into more than 25 languages, including Swedish [28]. The latest version, version three, contains the following seven sections: requirements at work, the organization and content of the work, relationships and leadership, interactions between the individual and their work, social capital, health and well-being and abusive behaviour [29]. The internal consistency (Cronbach's alpha) was above 0.70 for all scales, except for the two-item scale for Quality in Work (0.69). Most dimensions had low floor and ceiling effects [28].

COPSOQ items have 5 response options on Likert-type scales, e.g., from a very low to a very high extent or never to always. All versions have undergone extensive psychometric testing, and the previous version, version 2, was also tested through an operationalization of the JD-R model [30]. The included areas from the COPSOQ 3 were compared to population benchmarks. One of the findings of that study was that a management style that promotes possibilities for professional development, provides the ability to influence the work situation and provides clarity regarding work expectations is particularly relevant for achieving job satisfaction and work engagement [30].

### Data analysis

Descriptive statistics (proportions and counts) were performed to present the study population demographics and professional characteristics. The comparison to the population benchmarks was presented as the mean with a 95% confidence interval. Confidence intervals that did not include their respective benchmarks were considered significantly different from the benchmark.

Since the study population was a convenience sample, a post hoc power analysis was performed in GPower 3.1, were most of the items had a power > 0.80 [31]. To study potential differences among the demographic and professional characteristics, pairwise t tests were used with Bonferroni adjustment for multiple comparisons with a significance level of 5%. IBM SPSS Statistics 27 was used for data processing and statistical calculation.

## Results

A total of 73 participants answered the COPSOQ 3, and 67 participants provided demographic data. All 73 questionnaires were included in the analysis, but internal data were missing for some variables. All 73 completed questionnaires with few internal missing data were consider, and we did not exclude any participants. Table 1 presents the specialist nurses’ surgical care demographics (internal missing data are not shown for the specific variables).

**Table 1:** Demographics (*n* = 67)

Demographics %(n)				
**Sex**				
Females	97.0%	(64)		
Males	3.0%	(2)		
**Age**				
Younger than 35 years	30.3%	(20)		
35 to 45 years	45.5%	(30)		
Older than 45 years	24.2%	(16)		
**Marital Status**				
Single	11.9%	(8)		
Married	53.7%	(36)		
Partner	34.3%	(23)		
**Children**			**Children at home**	
Has no children	23.9%	(16)	-	
1	13.4%	(9)	77.8%	(7)
2	41.8%	(28)	85.7%	(24)
3 or more	20.9%	(14)	85.7%	(12)

The included professionals’ characteristics are shown in Table 2. The majority had a one-year master’s degree as well as many years of clinical experience as a registered nurse (> 10 years). Most participants (83.6%) had only one workplace as a specialist nurse in surgical care, and half of the participants had a manager with further education at an advanced level or specialist training in nursing.

**Table 2:** Professional characteristics (*n* = 73)

Profession %(n)		
**Specialist nurse in surgical care**		
Professional degree	3.0%	(2)
Master's degree	91.0%	(61)
Doctorate	6.0%	(4)
**Type of surgical care**		
Inpatient care	19.4%	(13)
Outpatient care	65.7%	(44)
Both/Other	14.9%	(10)
**Has a boss with further education or specialist training in nursing**		
Yes	40.3%	(27)
No	50.7%	(34)
Do not know	9.0%	(6)
**Has colleagues with further education or specialist training in nursing**		
Yes	85.1%	(57)
No	14.9%	(10)
**Experience as registered nurse (years)**		
< 10	33.3%	(21)
10–20	46.0%	(29)
> 20	20.6%	(13)
**Number of workplaces as a registered nurse**		
1	36.4%	(24)
2	36.4%	(24)
3 or more	27.3%	(18)
**Experience as a specialist nurse (years)**		
< 2	22.7%	(15)
2–5	37.9%	(25)
> 5	39.4%	(26)
**Number of workplaces as a specialist nurse in surgical care**		
1	83.6%	(56)
2	11.9%	(8)
3 or more	4.5%	(3)
**Working as specialist nurse in surgical care at current workplace (years)**		
< 3	46.8%	(29)
3–10	38.7%	(24)
> 10	14.5%	(9)

The following four areas in the COPSOQ were identified as factors related to success: quality of leadership, variation of work, meaning of work and work engagement, which were rated with higher mean scores compared to population benchmarks. In addition, job insecurity was indicated by a much lower mean score, which was desirable for this specific question.

**Fig. 1:**
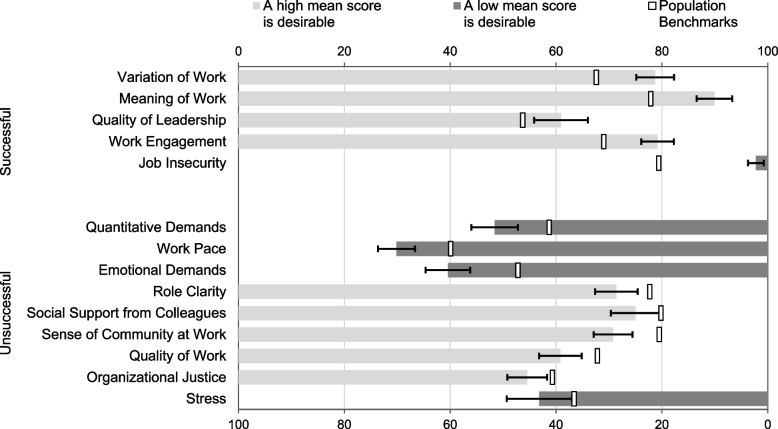
Successful and unsuccessful factors in relation to the Swedish population benchmarks

Although the study aimed to investigate factors related to success and less success in relation to the Swedish population benchmarks, nine domains were identified as factors related to less success in the analysis (see Fig. 1).

The mean scale scores in relation to the population benchmarks for workers in Sweden are presented in Table 3.

**Table 3:** Mean scale scores in relation to the population benchmarks for workers in Sweden

	At these scales, a **high mean score** is desirable	At these scales, a **low mean score** is desirable	**Population Benchmarks**^**a**^
**Requirements for work**			
Quantitative Demands		51.6 (47.2–56.0) −	40.9
Work Pace		70.1 (66.7–73.6) −	59.5
Emotional Demands		60.4 (56.2–64.7) −	46.8
**Work organization and content**			
Influence	49.5 (45.4–53.7)		50.2
Possibilities for Development	74.1 (70.0–78.1)		70.4
Variation of Work	78.7 (75.2–82.3) +		68.0
Meaning of Work	89.9 (86.6–93.3) +		78.3
**Relationships and leadership**			
Predictability	61.0 (57.0–65.0)		60.2
Recognition	67.0 (61.8–72.1)		65.6
Role Clarity	71.4 (67.4–75.4) −		78.1
Role Conflicts		45.1 (41.0–49.3)	42.2
Quality of Leadership	60.9 (55.9–66.0) +		54.1
Social Support from Supervisors	69.8 (63.9–75.7)		75.3
Social Support from Colleagues	75.0 (70.3–79.7) −		80.2
Sense of Community at Work	70.8 (67.1–74.4) −		79.9
**Interaction between the individual and their work**			
Commitment to the Workplace	62.5 (58.5–66.5)		64.7
Work Engagement	79.2 (76.1–82.2) +		69.4
Job Insecurity		2.2 (0.8–3.7) +	20.2
Insecurity over Working Conditions		27.8 (21.7–33.9)	24.9
Quality of Work	60.8 (56.8–64.9) −		68.2
Job Satisfaction	63.3 (59.1–67.6)		64.4
Work Life Conflict		43.7 (37.1–50.3)	39.7
**Social capital**			
Horizontal Trust	70.5 (66.7–74.2)		71.3
Vertical Trust	67.2 (63.3–71.2)		69.3
Organizational Justice	54.5 (50.8–58.3) −		59.7
**Health and well-being**			
Self-Rated Health	60.8 (54.8–66.8)		61.3
Burnout		41.6 (35.1–48.1)	36.0
Stress		43.2 (37.0–49.3) −	36.2

+ Significantly better than the population Benchmark

-Significantly worse than the population Benchmark

^a^For 25–65-year-old workers in Sweden (Berthelsen, 2020)

To answer the research question of significant differences between the demographic variables and factors, pairwise t tests were used. The results also showed significant differences between the participants who had and those who did not have a manager with specialist training in nursing; the latter group, to a greater extent, perceived job insecurity (*p* = 0.036) and insecurity over their working conditions (*p* = 0.021). *P* values are not shown for items that were tested and did not show significant differences at a 5% significance level.

The results showed that role clarity significantly differed between nurses aged 35 to 45 years and specialist nurses in surgical care who were older than 45 years (*p* = 0.019), and there was also an observed difference (not statically significant) between nurses who were younger than 35 years and nurses who were older than 45 years. This can be interpreted as an older age leading to a clearer professional role.

Higher scores were also found for variation of work (*p* = 0.039), role clarity (*p* = 0.016) and sense of community at work (*p* = 0.012) between specialist nurses in surgical care who had colleagues with specialist education and those who did not.

Differences between RNs who had worked in a single and three or more workplaces were found. RNs who had worked in three or more workplaces provided lower scores for possibilities for development (*p* = 0.016), recognition (*p* = 0.037), social support from colleagues (*p* = 0.013), and organizational justice (*p* = 0.040).

Furthermore, RNs who had worked in two workplaces provided higher scores for possibilities for development (*p* = 0.046) and quality of leadership (*p* = 0.30) than those who had worked in three or more workplaces, and lower scores for self-rated health than those who had worked in a single workplace (*p* = 0.020).

Finally, a higher score on organizational justice (*p* = 0.043) was found for RNs who had worked in a single workplace as a specialized nurse in surgical care compared to those who had worked in three or more workplaces.

## Discussion

This study provides valuable knowledge on specialist nurses in surgical care and their perspectives of organizational and social work environments in the surgical care context. The main results show that quality of leadership is important for the professional development of specialist nurses in surgical care. The five domains that were identified as being related to success compared to the population benchmarks were quality of leadership, variation of work, meaning of work and work engagement as well as low job insecurity. There was a correlation between nurse managers with a lower level of nursing education and uncertainty about working conditions. Previous studies have shown the importance of excellent leadership and a nurse's level of education [32, 33]. It has been suggested that high-quality leadership may reduce nurses’ job stress and intentions of leaving the field. Transformational and transactional leadership styles may reduce nurses’ job stress and intentions to leave the field, so nurse leaders can use a combination of transformational and transactional leadership to improve job satisfaction and the quality of nursing services. This means that nurse leaders can have an impact on improving job satisfaction, the quality of nursing and nurses’ education levels, which may impact patient mortality in surgical care [32, 33]. However, it is also important that nurses also take responsibility for their own situations and create good relationships with management.

Based on the results in the study, we interpret that improvement seems to include higher levels of nursing education for managers, which in turn may reduce the perception of insecure working conditions for specialist nurses in surgical care. To demonstrate a measurable outcome of developed nursing leadership, specialist nurses in surgical care must perceive that their efforts during and after graduation are valuable and that they are supported in their work. Magnet hospitals are characterized by a high level of competence in nursing leadership [17, 18], and nurse managers need to facilitate and enhance nurses’ use of evidence-based practices. Both managers and nurses need to have the necessary academic preparation, support and resources required for practices using evidence-based nursing.

It is a worrisome development that some hospitals and departments do not have nurse managers with nursing expertise and a bachelor’s degree. This can have both short- and long-term consequences for the quality of care, and strategic work is usually absent when nursing leaders have low education levels. Given these circumstances, it is hardly surprising that specialist nurses may lack the right conditions and support to provide high-quality surgical care.

High-quality leadership in nursing is necessary to obtain long-term profits to improve both the quality and efficiency of hospital care [34–36]. The lack of nurses with a higher education leads to a deterioration in the quality of care and missed nursing care, which may have serious consequences for patient safety [23–26]. Educating specialist nurses and then failing to provide the right conditions for them to provide high-quality surgical care due to a lack of competent leadership is a waste of resources, both from a health economic and a patient safety perspective.

Our results showed that specialist nurses in surgical care who had worked in three or more workplaces provided lower scores for possibilities for development, recognition and organizational justice. A higher score for organizational justice was found for those who had worked in a single workplace as a specialized nurse in surgical care compared to those who had worked in three or more workplaces. The imbalance between work requirements and work resources can, in accordance with the JD-R model, have organizational consequences, as this can lead to reduced motivation and professional development with the risk of specialist nurses in surgical care choosing to leave the workplace and/or profession [21, 22].

Strategic work seems to include managers with a higher nursing education level to prevent insecure professional working conditions. Health care organizations, including surgical clinics, often find it difficult to overcome the lack of competence in nursing, and there is a focus on "putting out fires" instead of long-term strategic work. This damage control does not provide opportunities for delivering the best evidence-based care, and the recruitment of new nurses is a costly and time-consuming procedure. Reversing a negative trend requires changes that create good conditions for high-quality surgical nursing and provide the opportunity for professional development.

The present study has strengths and limitations. A strength of this study is that the validated COPSOQ was used. The COPSOQ is a generic and theoretically based questionnaire that measures different aspects of the organizational and social work environment. The questionnaire has undergone psychometric testing and has been tested through an operationalization of the JD-R model [29]. The data in this study were compared to population benchmarks, which is also interpreted as a strength.

### Limitations

Limitations of the study are recognized. Firstly, the cross-sectional data and a strategic convenience sampling procedure may be interpreted as a limitation on the generalizability of the study. Secondly, the sample size may have affected the results. Due to the fact that this study was based on a convenience sample of 73 specialist nurses in surgical care and the data collection procedure. However, it is not exactly known what percentage of NFSK members are active in social media, so the response rate was difficult to exactly calculate for the research team. Since the study population was a convenience sample, a post hoc power analysis was performed, and the study was adequately powered. We aimed for a power > 0.80, and the most items were > 0.80. However, the study was conducted with specialist nurses in surgical care in a variety of hospitals in Sweden. The lack of specialist nurses leads to a deterioration in the quality of care and missed nursing care, which may have serious consequences for patient safety [23–26].

## Conclusions

Our results showed that specialist nurses in surgical care who had worked in a single workplace provided a higher score for organizational justice compared to those who had worked in three or more workplaces. The imbalance between work requirements and work resources may have organizational consequences, as this can lead to reduced motivation and professional development with a risk that specialist nurses in surgical care choose to leave the workplace and/or the profession. There was a correlation between nurse managers with a lower level of nursing education and uncertainty about working conditions, and the quality of leadership is important for professional development in specialist nurses in surgical care. The research implications are a strategic work for policy to recruit managers with a higher nursing education level to prevent insecure professional working conditions.

## Data Availability

The data generated and analyzed during the current study are not publicly available due to the confidentiality agreement with the participants. The data is however available from the corresponding author on reasonable request.
